# Limited Mitochondrial Permeabilization Causes DNA Damage and Genomic Instability in the Absence of Cell Death

**DOI:** 10.1016/j.molcel.2015.05.028

**Published:** 2015-06-04

**Authors:** Gabriel Ichim, Jonathan Lopez, Shafiq U. Ahmed, Nathiya Muthalagu, Evangelos Giampazolias, M. Eugenia Delgado, Martina Haller, Joel S. Riley, Susan M. Mason, Dimitris Athineos, Melissa J. Parsons, Bert van de Kooij, Lisa Bouchier-Hayes, Anthony J. Chalmers, Rogier W. Rooswinkel, Andrew Oberst, Karen Blyth, Markus Rehm, Daniel J. Murphy, Stephen W.G. Tait

(Molecular Cell 57, 860–872; March 5, 2015)

In the above article, the authors inadvertently presented the long exposure of caspase-3 input instead of caspase-7 (Figure 2D). The long exposure of caspase-7 input is now shown. The authors apologize for any confusion that this may have caused.

